# What is the impact of a clinically related readmission measure on the assessment of hospital performance?

**DOI:** 10.1186/s12913-017-2742-x

**Published:** 2017-11-28

**Authors:** Roger K. Khouri, Hechuan Hou, Apoorv Dhir, Juan J. Andino, James M. Dupree, David C. Miller, Chad Ellimoottil

**Affiliations:** 1 Dow Division of Health Services Research, Department of Urology, North Campus Research Complex (NCRC), 2800 Plymouth Road, Building 16, Ann Arbor, MI 48109 USA; 2 Michigan Value Collaborative, North Campus Research Complex (NCRC), 2800 Plymouth Road, Building 16, Ann Arbor, MI 48109 USA; 3 University of Michigan’s Ross School of Business, 701 Tappan Ave, Ann Arbor, MI 48109 USA; 4 U-M Institute for Healthcare Policy & Innovation, North Campus Research Complex (NCRC), 2800 Plymouth Rd, Bldg 16, 1st Floor, Room 100S, Ann Arbor, MI 48109-2800 USA

**Keywords:** Hospital admission, Hospital utilization, Medicare, Cost effectiveness, Billing and compliance, Readmission, Hospital readmission reduction program, Clinically related readmissions

## Abstract

**Background:**

The Hospital Readmission Reduction Program (HRRP) penalizes hospitals for high all-cause unplanned readmission rates. Many have expressed concern that hospitals serving patient populations with more comorbidities, lower incomes, and worse self-reported health status may be disproportionately penalized by readmissions that are not clinically related to the index admission. The impact of including clinically unrelated readmissions on hospital performance is largely unknown. We sought to determine if a clinically related readmission measure would significantly alter the assessment of hospital performance.

**Methods:**

We analyzed Medicare claims for beneficiaries in Michigan admitted for pneumonia and joint replacement from 2011 to 2013. We compared each hospital’s 30-day readmission rate using specifications from the HRRP’s all-cause unplanned readmission measure to values calculated using a clinically related readmission measure.

**Results:**

We found that the mean 30-day readmission rates were lower when calculated using the clinically related readmission measure (joint replacement: all-cause 5.8%, clinically related 4.9%, *p* < 0.001; pneumonia: all cause 12.5%, clinically related 11.3%, *p* < 0.001)). The correlation of hospital ranks using both methods was strong (joint replacement: 0.95 (*p* < 0.001), pneumonia: 0.90 (*p* < 0.001)).

**Conclusions:**

Our findings suggest that, while greater specificity may be achieved with a clinically related measure, clinically unrelated readmissions may not impact hospital performance in the HRRP.

**Electronic supplementary material:**

The online version of this article (10.1186/s12913-017-2742-x) contains supplementary material, which is available to authorized users.

## Background

In an effort to promote high-value care and reduce healthcare expenditures, the Centers for Medicare and Medicaid Services (CMS) implemented the Hospital Readmission Reduction Program (HRRP) in 2012 [1]. Through the program, CMS reduces all Medicare payments to hospitals with an excess number of rehospitalizations within 30 days after an acute inpatient stay for acute myocardial infarction, congestive heart failure, pneumonia, joint replacement, and chronic obstructive pulmonary disease. A hospital’s readmission rate is considered “excessive” if it exceeds what would be expected for an average hospital with a similar patient population. Hospitals with an excessive number of readmissions may have all of their Medicare payments reduced by up to 3%. In fiscal year 2015, hospitals were penalized by an aggregate sum of $424 million, with only 10% of hospitals accounting for nearly 50% of the penalities [1].Ø

A major source of criticism of the HRRP is that hospitals that serve patient populations with more comorbidities, lower incomes, and worse self-reported health status may be disproportionately penalized by the specifications of the program [2–5]. This unintended consequence may be partly explained by the fact that the readmission measure used in HRRP captures readmissions from nearly all causes, regardless of the initial reason for admission. For example, if a patient is admitted for a joint replacement and is rehospitalized within 30 days for diverticulitis, the patient’s readmission is counted against the hospital. Accordingly, while a hospital may be particularly adept at preventing joint replacement-related readmissions, they may still have a high readmission rate if the patient population that they serve has a high prevalence of comorbidities or has poor access to preventative care. It is possible that the use of a diagnosis-specific readmission measure would mitigate this unintended consequence.

In this context, we sought to determine if a clinically related readmission measure would significantly alter the assessment of hospital performance. We compared hospital performance for two conditions in the HRRP (joint replacement and pneumonia) using CMS’ all-cause unplanned readmission measure versus a clinically related readmission measure developed by the Michigan Value Collaborative (MVC). Specifically, we used Michigan Medicare data to answer three key questions. First, how do hospital readmission rates compare when calculated using an all-cause unplanned readmission measure and a clinically related readmission measure? Second, do hospital rankings change when a different measure is used? Finally, what are common diagnoses that are included by the all-cause unplanned measure but excluded by the clinically related measure? By virtue of this approach, our findings will provide policymakers, hospital administrators, and physicians with more nuanced insight regarding the impact of unrelated readmissions on hospital performance in HRRP.

## Methods

### Patient population and data sources

We analyzed Medicare claims for beneficiaries in the state of Michigan who had index admissions for lower extremity joint replacement or pneumonia from January 1, 2011 through October 1, 2013 to one of 63 hospitals that participate in the MVC [6]. All 63 hospitals enrolled in the MVC at the beginning of the study were included. The MVC is a Blue Cross Blue Shield of Michigan-funded initiative that provides commercial and Medicare data to hospitals.

Patients were identified using International Classification of Diseases, Ninth Revision (ICD-9) diagnosis and procedure codes identical to those in the HRRP [7]. We excluded beneficiaries that were not continuously enrolled in Medicare Part A and B, died during the index admission, had HMO coverage, or were entitled to Medicare because of end-stage renal disease or disability. In addition, for joint replacement admissions, we excluded patients who had a primary diagnosis of hip fracture (12.3% of episodes). The Institutional Review Board of our health system deemed this study exempt from review.

### Calculating all-cause unplanned and clinically related readmission rates

We calculated two 30-day readmission rates for each hospital for each condition. First, we calculated each hospital’s 30-day all-cause unplanned readmission rate; this is the readmission rate used by CMS for the HRRP. To do this, we first identified all readmissions and excluded those that are classified as “planned” by the published CMS algorithms for pneumonia and joint replacement [7]. We did not perform any risk adjustment calculations when measuring all-cause unplanned readmissions. Planned readmissions are defined by specific ICD-9 diagnosis and procedure codes. Planned readmissions are non-acute readmissions for scheduled procedures; they also include maintenance chemotherapy/immunotherapy, obstetrical delivery, rehabilitation, and transplant surgery.

We then calculated each hospital’s 30-day clinically related readmission rate. To do this, we used algorithms developed by the MVC’s panel of clinical experts. In the MVC algorithm, the first and second ICD-9 diagnosis codes of the inpatient claim for any readmission are compared against the MVC list of “clinically related” ICD-9 diagnosis codes. For example, a readmission claim for knee replacement may have a primary or secondary ICD-9 diagnosis code of “719.96: Hemarthrosis, lower leg”. This claim would be considered related to the joint replacement. In contrast, ICD-9 diagnosis code 209.12: “Malignant carcinoid tumor of the cecum” would be considered unrelated to the joint replacement and would not be included in the clinically related readmission rate. Examples of readmission claims considered clinically related to joint replacement and pneumonia are listed in Additional file 1. Additional file 2 includes the full list of clinically related readmission claims for each condition.

### Comparing readmission rates

We compared the all-cause unplanned readmission rate to the clinically related readmission rate in several ways. First, we compared the readmission rates at the hospital-level using a paired *t*-test. Second, we ranked hospitals using both the all-cause unplanned and clinically related readmission rates and calculated the correlation between these two rankings. Third, to better understand the causes of non-clinically-related readmissions, we examined the principal diagnosis codes associated with the readmissions that were included by the all-cause unplanned readmission measure but excluded by the clinically related readmission measure. Finally, we examined readmissions that were included by the clinically related readmission measure but excluded by the all-cause unplanned readmission measure because they were considered “planned” by the CMS algorithm.

### Sensitivity analyses

In addition to the overall comparison, we performed two sensitivity analyses. First, we examined the mean difference in hospital rank correlation using 90-day readmission rates. Second, we stratified hospitals into quartiles based on percentage of Medicaid admissions. Theses sensitivity analyses were performed to understand whether correlation rates would be different if a longer time window was used or if hospitals were grouped by their patient population. All analyses were performed using statistical software (STATA 13/SE, College Station, TX) at the 5% significance level.

## Results

We identified 26,369 index admissions for joint replacement surgery, and 18,895 index admissions for pneumonia in 63 Michigan hospitals. Among this group, we identified 1499 total readmissions within 30 days of joint replacement procedures and 3241 total readmissions within 30 days of a hospitalization for pneumonia. Table 1 presents the data averaged per hospital with standard deviations and percentiles.

**Table 1:** Readmissions per hospital

	Joint Replacement	Pneumonia	Total
Index Admissions Per Hospital	419 +/− 368 (337, 121–835)	300 +/− 169 (266, 113–555)	718 +/− 505 (593, 249–1315)
Total Readmissions Per Hospital	23.8 +/− 23.9 (17, 6–42)	40.2 +/− 23.1 (39, 12–76)	64.0 +/− 43.2 (60, 20–114)
All-Cause Unplanned Readmissions Per Hospital	23.4 +/− 23.5 (16, 6–42)	37.4 +/− 21.4 (35, 11–68)	60.9 +/− 41.4 (55, 19–108)
Clinically-Related Readmissions Per Hospital	19.7 +/− 20.5 (13, 5–34)	33.6 +/− 19.2 (32, 11–63)	53.3 +/− 36.1 (48, 18–95)

Data are presented as mean +/− standard deviation (median, 80% range)

The mean all-cause unplanned readmission rate was 5.8% (range 1.4%–11.4% across hospitals) for joint replacement and 12.5% (range 4.4–20.7%) for pneumonia. The mean clinically related readmission rates for joint replacement and pneumonia were 4.9% (range 1.4%–9.6%) and 11.3% (range 4.4%–20.5%), respectively.

Hospitals generally had lower rates of readmission with MVC’s clinically related definition versus CMS’ all-cause unplanned readmission algorithm. For joint replacement, 100% of hospitals had an equal or lower clinically related readmission rate. For pneumonia, 90% of hospitals had an equal or lower clinically related readmission rate. The mean difference between the all-cause unplanned and the clinically related readmission rates was 0.9% (range 0% to 2.8% across all 63 hospitals) for joint replacement and 1.2% (range − 1.8%-6.1%) for pneumonia. The mean difference between these two rates was statistically significant for both conditions (*p* < 0.001).

**Fig. 1:**
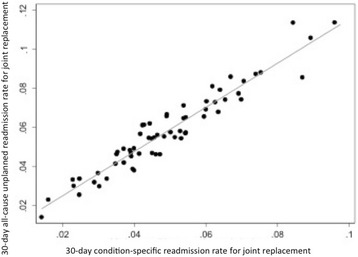
Hospital-level comparison between 30-day all-cause unplanned readmission rate for joint replacement and 30-day condition-specific readmission rate for joint replacement. The rank correlation between both methods was 0.95 (*p* < 0.001)

**Fig. 2:**
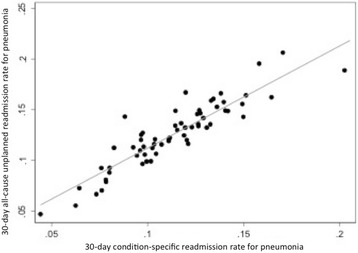
Hospital-level comparison between 30-day all-cause unplanned readmission rate for pneumonia and 30-day condition-specific readmission rate for pneumonia. The rank correlation between both methods was 0.90 (*p* < 0.001)

The rank correlation between both methods was 0.95 (*p* < 0.001) for joint replacement and 0.90 (*p* < 0.001) for pneumonia (Figs. 1 and 2). The 90-day sensitivity analysis shows that the correlation of hospital ranks using both methods was also strong (joint replacement: 0.95 (*p* < 0.001), pneumonia: 0.89 (*p* < 0.001)). We found no substantive difference in the correlation rates when we stratified hospitals by quartile of Medicaid admissions for joint replacement. However, for pneumonia, the correlation between these two rates was lower (albeit still fairly strong) among hospitals in the highest quartile for Medicaid admissions (0.79, *p* < 0.001).

There were 247 (16%) 30-day readmissions for joint replacement and 419 (13%) 30-day readmissions for pneumonia that were included in the CMS all-cause readmission measure but excluded by the MVC clinically related readmission measure (Table 2). For both conditions, atrial fibrillation was the most common readmission diagnosis that was not considered to be clinically related (Table 3).

**Table 2:** Agreement between the all-cause unplanned readmission measure and clinically related readmission measure

Joint Replacement			Pneumonia		
All-cause unplanned readmission measure	Clinically-related readmission measure		All-cause unplanned readmission measure	Clinically-related readmission measure	
	Included	Excluded		Included	Excluded
Included	1229 (82%)	247 (16%)	Included	2626 (81%)	419 (13%)
Excluded	13 (1%)	10 (1%)	Excluded	119 (4%)	77 (2%)

Source: Michigan Medicare Data, 2011–2013

**Table 3:** Top ten primary diagnoses for 30-day readmissions that are included by the CMS all-cause readmission measure but excluded by the MVC clinically related measure

Joint replacement			Pneumonia		
Primary diagnosis	Frequency	Percent	Primary diagnosis	Frequency	Percent
Atrial fibrillation	27	11.1%	Atrial fibrillation	25	6.1%
Diverticulitis without hemorrhage	9	3.7%	Chest pain	15	3.7%
Chest pain	8	3.3%	CHF	13	3.2%
Chest pain (not specified)	6	2.5%	Cardiac dysrhythmias	12	2.9%
Constipation	6	2.5%	Alzheimer’s disease	11	2.7%
Intestinal obstruction	6	2.5%	Diverticulitis with hemorrhage	8	2.0%
Syncope and collapse	6	2.5%	Intestinal obstruction	7	1.7%
Blood in stool	5	2.1%	Orthostatic hypotension	7	1.7%
Cardiac dysrhythmias	5	2.1%	Diabetes mellitus II	6	1.5%
Intestinal adhesions with obstruction	5	2.1%	Diverticulitis without hemorrhage	6	1.5%
Other	164	66.3%	Other	309	73.7%

Percentages are based on total number of 30-day all-cause readmissions included by the all-cause readmission measure but excluded by the clinically related measure for joint replacement (*n* = 247) and pneumonia (*n* = 419). Source: Michigan Medicare Data, 2011–2013

Thirteen 30-day readmissions (0.9%) for joint replacement and 119 (4%) 30-day readmissions for pneumonia were classified as “planned” readmissions using the CMS measure (i.e., excluded from the all-cause unplanned readmission rate calculation) but were included by MVC’s clinically related readmission measure. Among these cases, 62% and 45% were considered emergent (by source of admission code or the presence of emergency room charges) for joint replacement and pneumonia, respectively.

## Discussion

For joint replacement and pneumonia, the clinically related readmission rate is marginally lower than the all-cause unplanned readmission rate. For individual hospitals, there was a strong correlation between the two measures. The CMS’ HRRP currently holds hospitals accountable for all-cause 30-day readmissions, regardless of whether or not they are clinically related to the index admission. While this might seem problematic, our study suggests that relatively few non-clinically-related admissions occur, and the difference in readmission measures will likely have a minimal impact on the assessment of hospital performance.

Our primary finding of a strong correlation between the all-cause and clinically related readmission rate is consistent with observations regarding the clinical epidemiology of readmissions after hospitalizations for joint replacement and pneumonia. For joint replacement, 30-day readmissions are relatively infrequent (about 5%), making the impact of different definitions predictably small. For pneumonia, Table 3 demonstrates that it is often difficult to determine whether or not the readmission is clinically related to the index admission. For example, atrial fibrillation was the most common non-clinically-related readmission diagnosis associated with pneumonia in the present study; however, atrial fibrillation is a risk factor for pneumonia and vice versa [8, 9]. Despite this established relationship between pneumonia and atrial fibrillation, the MVC algorithm does not consider readmissions for atrial fibrillation to be clinically related to an index admission for pneumonia for several reasons. Pneumonia is only a risk factor for atrial fibrillation during an ongoing infection; there is no evidence that a recent history of pneumonia is a risk factor for atrial fibrillation. Additionally, a patient discharged after an admission for pneumonia who is readmitted to the hospital within 30 days with a primary diagnosis of atrial fibrillation most likely has a primary cardiac issue that is not clinically related to the pneumonia or its treatment. This example demonstrates the difficulty of categorically determining which readmissions are clinically related to the index admission without analyzing each readmission individually. This is a major limitation to implementing a clinically-related readmission measure.

Previous studies comparing all-cause readmissions, potentially preventable readmissions, and condition-specific readmissions have found varying degrees of correlation between readmission measures [10–13]. Differences in hospital types, admitting diagnoses, and readmission algorithms studied might explain this variation in correlation. Additional studies are needed to further clarify the effects of different readmission algorithms on hospital performance in specific situations.

Our study has several limitations. First, we focused our analyses on only two conditions. As such, our findings may not be generalizable to all (current and future) conditions in the HRRP. As there have been no previous studies using the MVC clinically-related readmission algorithm, we elected to focus on only two conditions for this first study. The findings from this study now provide a basis for future studies on all of the conditions included in the HRRP. Second, the clinically related readmission measure developed by MVC has not undergone external validation. However, the measure has face validity after being reviewed by clinical experts in these areas. Third, interpretation of our data is complicated by the fact that “planned” readmissions were included in the clinically-related readmission measure and not in the all-cause readmission measure. However, the primary purpose of the study is to determine the effect of excluding non-clinically-related readmissions. Thus, any readmission determined to be clinically-related to the index admission should be included in the MVC clinically-related readmission measure, regardless of whether or not it was planned. Additionally, only a small percentage of clinically-related readmissions were considered planned (0.9% for pneumonia and 4% for joint replacement) by the CMS planned/unplanned algorithm, and the majority of these “planned” readmissions were found to be emergent. Therefore, a very small percentage of clinically related admissions were actually planned, and this small percentage would not have a significant effect on the overall study findings. Finally, our analysis is limited to hospitals in Michigan, so our findings may not be generalizable nationally. However, Michigan is the tenth largest state by population and has a wide range of socioeconomic statuses, so we do not expect there to be significant differences between our data and the rest of the country.

These limitations notwithstanding, our results have important implications for several key stakeholders in this area, including policy makers, systems leaders, administrators, and clinicians. A large body of evidence shows that readmissions are driven by factors independent of the medical condition of the patient [4, 14–16]. However, the strong correlation between CMS’ all-cause unplanned readmission measure and MVC’s clinically related readmission measure suggests that the HRRP’s use of an all-cause unplanned readmission measure may not unfairly penalize hospitals who care for high-need patients [17]. Hospitals that serve high-need populations will likely perform poorly, regardless of which readmission measure is used. Additionally, the fact that most readmissions were clinically related to the index admission suggests that efforts to reduce readmission rates should focus on initiatives clinically related to the index admission (e.g., improved discharge planning, close post-discharge follow up) rather than general health.

## Conclusions

Taken together, our findings suggest that use of a clinically related algorithm for identifying related readmissions, as opposed to CMS’ all-cause unplanned readmission measure, will not significantly change the assessment of hospital performance in the HRRP for joint replacement and pneumonia. An important next step is to determine whether the same relationship between all-cause unplanned and clinically related readmission measures exists for other conditions. In addition, to avoid unfairly penalizing hospitals, efforts should continue to identify other root causes of readmissions (e.g., cognitive impairment, substance use, poor access to transportation and social support) and to develop methodologies that accounts for these important concerns [16, 18–20]. In the end, the value of any readmission program should be to identify and respond to signals of poor quality without penalizing hospitals for events that are out of their control.

## Additional files

Additional file 1: Examples of ICD-9 codes considered clinically related to index admissions for pneumonia, joint replacement, and all conditions. (DOCX 65 kb)

Additional file 2: Codes clinically related to index admissions for all conditions. Codes considered to be clinically related to index admissions for hip replacement. Codes considered to be clinically related to index admissions for knee replacement. Codes considered to be clinically related to index admissions for pneumonia. (XLSX 69 kb)

## Abbreviations

CMS: Center for Medicare & Medicaid Services
HRRP: Hospital Readmission Reduction Program
ICD-9: International Classification of Disease, ninth revision
MVC: Michigan Value Collaborative
