# Supplementary material for: What is the impact of a clinically related readmission measure on the assessment of hospital performance?
Source: BMC Health Serv Res. 2017 Nov 28;17:781. doi: 10.1186/s12913-017-2742-x (PMC5704581; doi:10.1186/s12913-017-2742-x)
Supplement: Supplementary file 1 — Examples of ICD-9 codes considered clinically related to index admissions for pneumonia, joint replacement, and all conditions. (DOCX 65 kb) [file 12913_2017_2742_MOESM1_ESM.docx]

**Appendix 1**. Examples of ICD-9 codes considered clinically related to index admissions for pneumonia, joint replacement, and all conditions.

| Joint Replacement | Pneumonia | All Conditions |
| --- | --- | --- |
| Cellulitis and Abscess of Leg (6826) | Septicemia (038) | Acute cystitis (5950) |
| Unspecified Infective Arthritis, Pelvic Region and Thigh (71195) | Disorders of Fluid Electrolyte and Acid-Base Balance (276) | Thrombocytopenia, unspecified (04109) |
| Unspecified Chondrocalcinosis, Pelvic Region and Thigh (71235) | Blood Stream Infection Due to Central Venous Catheter (99932) | Occlusion and Stenosis of Carotid Artery (43310) |
| Unspecified Monoarthritis, Pelvic Region and Thigh (71665) | Chronic Pulmonary Heart Disease (416) | Intestinal Infection due to Clostridium Difficile (00845) |
| Effusion of Joint, Lower Leg (71906) | Acute Myocardial Infarction (410) | Septic Shock (78552) |
